# Effect of Treating Eggs with Coenzyme Q10 (CoQ10) on Growth Variables, Histomorphometry, and Antioxidant Capacity in Red Tilapia (*Oreochromis aureus × Oreochromis mossambicus*) Larvae

**DOI:** 10.3390/ani12172219

**Published:** 2022-08-29

**Authors:** Mona M. Mourad, Shimaa A. Shahin, Ibrahim T. El-Ratel, Mohammed F. El Basuini

**Affiliations:** 1 National Institute of Oceanography and Fisheries (NIOF), Cairo 11516, Egypt; 2 Animal and Fish Production Department, Faculty of Agriculture-Saba Basha, Alexandria University, Alexandria 21531, Egypt; 3 Department of Poultry Production, Faculty of Agriculture, Damietta University, Damietta 34517, Egypt; 4 Faculty of Desert Agriculture, King Salman International University, El Tor 46612, Egypt; 5 Department of Animal Production, Faculty of Agriculture, Tanta University, Tanta 31527, Egypt

**Keywords:** CoQ10, Red tilapia, larvae, antioxidants, gut histology

## Abstract

**Simple Summary:**

Obtaining superior eggs and larvae is the first stage toward sustainable aquaculture. The production of aquatic animal larvae is frequently obstructed by high death rates, and water-soluble stimulants are suggested to be more effective in the early stages, e.g., eggs and yolk larvae stage. A limited number of studies exist on the use of coenzyme Q10 (CoQ10) in aquaculture, particularly as a dietary supplement, and to the authors’ knowledge, there are insufficient studies on the application of CoQ10 as a water treatment. Therefore, this study attempted to evaluate the possible impacts of the exposure of red tilapia eggs to CoQ10 in hatching water.

**Abstract:**

Red tilapia eggs one day post fertilization (dpf) were exposed to coenzyme Q10 (CoQ10) at rates of 0, 5, and 10 mg/L for control, treatment 2 (C5), and treatment 3 (C10), respectively, without exchanging water and until the larval mouth-opening stage. Fertilized eggs of red tilapia exposed to different concentrations of CoQ10 were hatched at rates (*p* > 0.05) between 38 to 54.67%. The yolk-sac diameter at the 2nd day post hatching (dph), ranged from 1.85 to 1.87 mm in depth and 1.63 to 1.88 mm in width and was not altered by the CoQ10 treatments. Similarly, red tilapia survival (*p* > 0.05) ranged from 22.67 to 32%. On 6 dph, a slight percentage (2.08%) of survived fishes exposed to high CoQ10 dose (C10) exhibited larval deformation in the form of an axial curvature of the spine in the abdominal and caudal region. Larvae displayed a normal structure of the esophagus folds in all fish groups, and larvae in the C5 group displayed the longest folds and widest muscularis layer, followed by fishes in the C10 group and the control. Red tilapia fry on 30 dph treated with CoQ10 possessed higher antioxidant potentials in terms of superoxide dismutase (SOD), catalase (CAT), and glutathione peroxidase (GPx) compared to the control. In conclusion, treating Red tilapia fertile eggs with 5 mg/L CoQ10 improves the growth, gut structure, and antioxidant efficiency of the produced larvae.

## 1. Introduction

With the recent population growth, a steady and durable source of high-quality protein has become vital, and aquaculture is seen as a reasonable answer for closing the gap between production and human consumption as well as restocking and conserving overfished stocks and threatened fish species [1,2]. Since the 1970s, aquaculture has made a substantial contribution to the production of seafood; nonetheless, the aquaculture business confronts several obstacles [3,4,5]. As extensive aquaculture grows, negative impacts and stressful conditions on fish and aquatic systems, such as larval death, pathogens outbreak, and water pollution, become increasingly common [6,7].

The success of aquaculture operations is controlled by different interconnected aspects, such as the cultured stock, which begins with larvae, and the aquatic environment. Obtaining superior eggs and larvae is the first stage toward sustainable aquaculture [8,9,10]. The production of fish larvae is frequently hampered by high death rates, which might be triggered by pathogens or environmental issues [10,11]. Aquatic species have a natural survival level of less than 1% until maturity, with substantial mortality rates in the early stages [12]. The embryo stage, the freshly hatched pre-feeding stage (yolk-feeding larvae), and the juvenile metamorphosis are all part of this vital phase [13]. Bacteria and fungi settle on the surface of fish eggs, and yolk-feeding larvae are vulnerable to this biofilm while their organs and functions are developing [10]. Altering feed is a key verified practice to modulate the growth and immune response of fry in the exogenous feeding stage but is not applicable in the early stages [14,15].

Water-soluble stimulants are suggested to be more effective in the early stages, e.g., eggs and yolk larvae stage [14,16]. Antibiotics have been used for centuries as remedies, growth promoters, and health stimulants, but due to widespread misuse and consequences, antibiotics have been outlawed in many countries [17]. Antibiotics and other synthetic compounds are progressively being substituted with environmentally acceptable alternatives [18,19,20].

Coenzyme Q10 (CoQ10), also known as ubiquinone or a super-vitamin (vitamin Q), is a potential candidate found normally in the biological system in most cells, where it is involved in the electron transport chain (ETC), ATP generation, and regenerated vitamin E, and it also has ability as an antioxidant [21,22,23,24,25]. Because CoQ10 is produced in inadequate quantities in the body, particularly during times of stress, it must be acquired from an external source [26]. Currently, there are many commercial CoQ10 products available, including both water- or lipid-soluble forms [21,27]. An insufficient number of studies exist on the use of CoQ10 in aquaculture, particularly as a dietary supplement [27,28], and to the authors’ knowledge, there are insufficient studies on the application of CoQ10 as a water treatment. Therefore, this study attempted to evaluate the possible impacts of the exposure of red tilapia eggs to CoQ10 in hatching water.

## 2. Materials and Methods

### 2.1. Broodstock and Eggs Maintenance

Red tilapia (*Oreochromis aureus* × *Oreochromis mossambicus*) eggs were from carefully selected broodstock in a ratio of 1 male:3 females [29,30] with an average standard length and weight of 15 ± 2 cm and 120 ± 10 g, respectively, using the natural spawning in a governmental aquaculture facility in finfish hatchery (GAFRD, west Alexandria, Egypt). Fertilized eggs at the late gastrula and pro-organogenesis stages were shifted from the broodstock mouth to transportation jars following a second repetitive cleaning with fresh water to remove any impurities. Non-fertilized, asymmetrical, vesicle-containing, or damaged eggs were removed. Immediately after arrival, eggs were placed in a semi-static glass aquaria system with 1 L of filtered saltwater, which was diluted at a rate of 5 ppt/h with de-chlorinated tap water until salinity reached 15 ppt with a pH of 7 while maintaining oxygen saturated (dissolved oxygen, DO = 6.87 ± 0.06 mg/L) by continuous aeration at room temperature. To avoid metabolic waste contamination, aquarium water was replenished every 24 h.

### 2.2. Experimental Exposure of Fertilized Eggs to CoQ10

Healthy, viable, fertilized eggs one day post fertilization (dpf) were placed in 5 L plastic jars (salinity = 15 ppt and pH = 7) at a rate of 20 eggs/L and implemented in triplicate for each treatment. During the treatment phase (3–4 days), CoQ10 (water-soluble CoQ10-BULK SUPPLEMENTS.COM ^®^) was dissolved in eggs’ hatching brackish water at rates of 0, 5, and 10 mg/L for control, treatment 2 (C5), and treatment 3 (C10), respectively, without exchanging water and until the larval mouth-opening stage (3 ± 1 dpf). The employed CoQ10 doses were suggested based on the previous work of Pasha and Moon [31]. Proper aeration was provided using air stones to prevent the eggs from collapsing at the bottom of the experimental jars. All of the tanks with embryos were kept at a constant temperature, salinity, dissolved oxygen, and photoperiod (28 °C, 15 ppt, 6.87 ± 0.06 mg/L, and a 10 h/14 h dark/light cycle, respectively) until hatching. Daily embryo and larvae mortalities were recorded, and the hatchability rate was estimated using the following equation:

Hatchability % = (No. of hatched eggs/No. of fertile eggs) × 100

The larvae were relocated to 20 L tanks once the larvae began exogenous feeding (3–4 dph) under the same controlled conditions without CoQ10 supplements. Following the treatment phase, all fish groups were given a commercial diet (58% CP: O.RANGE-INVE AQUACULTURE INC.) for 15 days, then another diet (48% CP: Nutra 0 Tilapia Feed-Skretting ^®^) for the same period until the experiment’s conclusion at 30 days post hatching (dph).

### 2.3. Growth Metrics

Standard length (Ls) and yolk sac diameter (depth and width) were determined using a stereomicroscope (OPTIKA, ISC366- Optika IS view, Ponteranica, Italy) and digitally photographed at a magnification of 0.7 with an Optika digital camera delivered with the analytic digital program Optika Vision Lite V.2.12 software. Survival rate was assessed using the following equation:

Survival rate (SR%) = (No. of survived larvae at 30 dph/No. of fertile eggs) × 100

### 2.4. Histological and Morphometric Analysis

Larvae were sampled at random (*n* = 3/tank) and fixed in 4% formalin, then dehydrated in a sorted ethanol sequence and embedded in paraffin. Slices were taken at 5 µm and stained with hematoxylin-eosin (H&E). Histological assessment was performed using light microscopy (LEICA, Leica Microsystems AG, Wetzlar, Germany) and digitally photographed at a magnification of 40× and 100× with Leica digital camera model D-LUX and administered using the ImageJ V.1.48 software.

### 2.5. Antioxidant Potential

Whole larval fish samples were homogenized in iced NaCl (0.86%) and centrifugated at 4 °C, 12000 rpm, for 15 min. The resulting supernatants were collected for colorimetric measurement of superoxide dismutase (SOD), catalase (CAT), and total glutathione peroxidase (GPx) at 550, 280, and 412 nm using a commercial detection kit (JianCheng, Nanjing, China) according to the factory protocol.

### 2.6. Statistical Analysis

Statistical evaluation of data (Means ± S.D. *n* = 3) was performed, plotted, and calculated by SPSS (Ver. 22, SPSS Inc., Chicago, IL, USA) following one-way ANOVA including the effect of coenzyme Q10 level. The normality and homogeneity of data were evaluated by Shapiro–Wilk and Levene tests, and means were checked for significance using the LSD test, and the level of significant difference was set at *p* < 0.05.

## 3. Results

### 3.1. Hatchability Rate, Growth Development, and Malformation Interpretation

**Figure 1:**
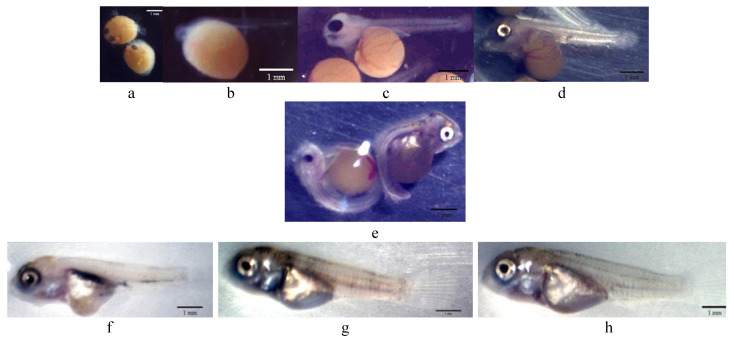
Morphological development of red tilapia (Scale bar 1 mm). (**a**) Egg; (**b**) 0 dph; (**c**) 1st dph; (**d**) 2nd dph; (**e**) 6th dph (malformation appeared in C10 group); (**f**–**h**) 30 dph red tilapia fry of the control, C5, and C10 groups, respectively.

Fertilized eggs of red tilapia (Figure 1a) exposed to different concentrations of CoQ10 were hatched at rates between 38 to 54.67% (Table 1). Eggs exposed to the high concentration of CoQ10 (C10) had an insignificant (*p* > 0.05) rise in the hatchability rate. The hatched larvae were loaded with a large valium of yolk sac out of its abdomen area that grants its endogenous feeding during the unopened-mouth larval phase (Figure 1b,c). The absorbance speed of the larval yolk sac was reflected in the larval development, in which the yolk-sac diameter (depth and width) was decreased with aging and increasing larval length. On the 1st day post hatch (1 dph), the yolk-sac width was significantly (*p* < 0.05) decreased with larval exposure to a low concentration of CoQ10 (C5) (Table 1). The yolk-sac diameter at 2nd dph (Figure 1d) ranged from 1.85 to 1.87 mm in depth and 1.63 to 1.88 mm in width and was not altered by the CoQ10 treatments.

Red tilapia survival ranged from 22.67 to 32% and was unaffected by CoQ10 exposure (Table 2). On 6 dph, a slight percentage (2.08%) of survived fishes exposed to a high CoQ10 dose (C10) exhibited larval deformation in the form of an axial curvature of the spine in the abdominal and caudal region. The curvatures were accompanied by a slight deformation of the yolk sac (Figure 1e). At 30 dph, fishes in the C5 group displayed higher final weight and total length than those in the C10 group, while no noticeable variations existed when compared to the control group (Table 2, Figure 1f–h).

### 3.2. Histological Assessment

**Figure 2:**
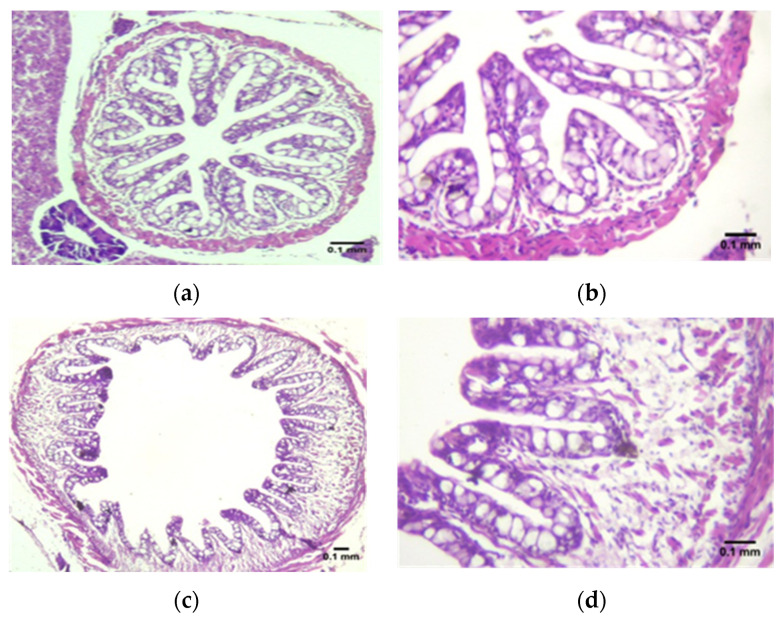

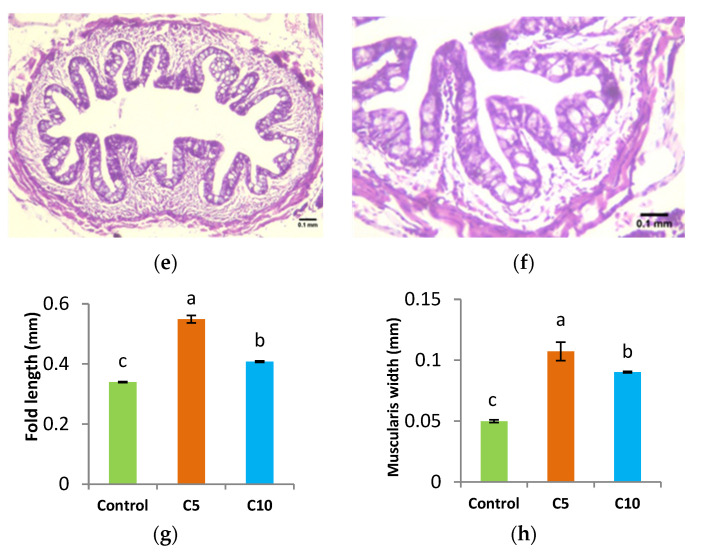
Histological variations (**a**–**f**) in the esophagus folds among trial groups. (**a**,**b**) Control; (**c**,**d**) C5; (**e**,**f**) C10 showing normal esophagus folds in all groups. Photos (**b**,**d**,**f**) are a magnification of (**a**,**c**,**e**), respectively (H&E staining, scale bar; 0.1 mm). Histograms (**g**,**h**) illustrate the variations in red tilapia esophagus folds length (**g**) and muscularis width (**h**) among the different experimental groups. Means with different letters are different.

**Figure 3:**
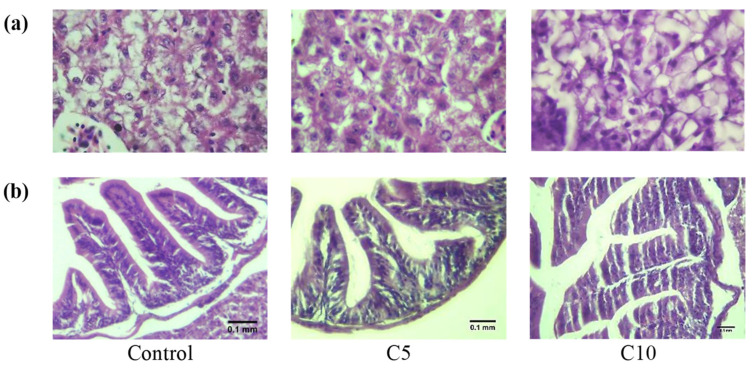
Histological sections show a normal structure of (**a**) liver and (**b**) intestine of red tilapia fish (H&E staining).

Histological examination showed a normal structure of the esophagus folds in all fish groups (Figure 2). The morphometric measurement of the esophagus folds demonstrated significant (*p* < 0.05) alterations in the length of the folds and thickness of the muscularis layer in all experimental groups. Fishes in the C5 group displayed the longest folds and widest muscularis layer, followed by fishes in the C10 group and the control. The histological examination of the stomach, intestine, and liver of red tilapia fish revealed a normal structure of epithelial cells in the lamina propria and submucosal layer with no sign of any inflammation (Figure 3).

### 3.3. Antioxidant Capacity

**Figure 4:**
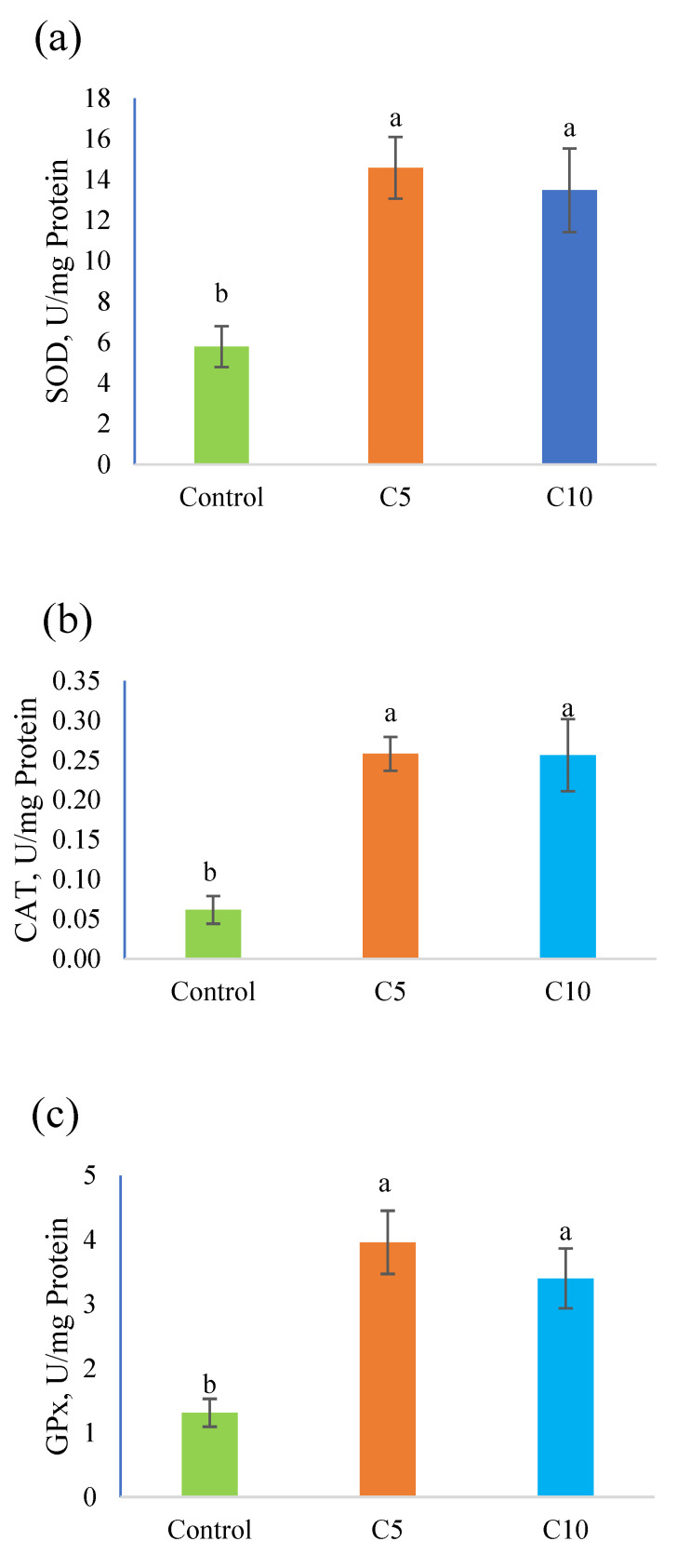
Antioxidant’s potential in red tilapia fry (30 dph). (**a**) Superoxide dismutase (SOD); (**b**) catalase (CAT); (**c**) glutathione peroxidase (GPx). Means with different letters are different.

Figure 4 shows the antioxidants potentials in red tilapia fry on 30 dph. Fishes treated with CoQ10 possessed higher antioxidant potentials in terms of SOD, CAT, and GPx compared to the control.

## 4. Discussion

Starting with high-quality eggs and larvae is one of the pillars of aquaculture sustainability [8,9,10]. Substantial mortality rates (more than 99 percent until maturity) thwart the production of fish larvae in the early stages, which could be caused by pathogens or environmental causes [10,11]. One of the most effective practices to obtain good-quality larvae is to treat embryos or fertilize eggs [32]. Eco-friendly natural techniques have drawn researchers’ interest as viable and effective alternatives to antibiotics [33,34,35,36]. Coenzyme Q10 (CoQ10) or ubiquinone is one probable model present naturally in the biological system and most cells with antioxidant potentials [24,25,37,38,39].

Results of hatchability rate (%) and yolk-sac diameter (depth and width, mm) of red tilapia larvae (Table 1) show no alteration in response to CoQ10 exposure, which reflects no alterations in the integrity of eggs or the development of embryos under the conditions of the trial. Previous studies indicate a change in hatching rate with the addition of antioxidants manifesting both positively [32] and negatively [40]. The difference in the results may be attributed to the difference in the experiment factors, such as the type and concentration of the antioxidant used and the quality of water.

Growth development results show a higher final weight and total length in the C5 group (Table 2), which decreased with minor deformation % in the C10 group (Figure 1e), and this may be linked to the hypercatabolic impact of CoQ10 at a high level for energy production. In this context, CoQ10′s function in the Krebs cycle makes it a critical piece of carbohydrate, protein, and lipid metabolism [21,22,23]. Furthermore, the indirect impact of CoQ10 on hormones function (insulin, glucagon, and cortisone) and/or vitamin E re-generation assists in altering the metabolic rate [24,25,37,38,41]. Changes in the histological composition of the gut support the overgrowth in the C5. The enhanced structure in CoQ10 groups may be associated with the anti-inflammatory merits of CoQ10 [42]. El Basuini et al. [28] linked enhanced growth with dietary CoQ10 supplements to the promoted digestive enzymes.

On the other hand, the negative effects of a high CoQ10 treatment (C10) could be the result of an imbalanced metabolism. According to this viewpoint, CoQ10′s pro-oxidant effects were reported at high doses [25]. In addition, the exogenous administration of CoQ10 at a high level may also have an impact on the structure and function of the liver due to the medication side effect on the liver brought on by excessive CoQ10 uptake that exceeds the capacity of adaptive response [43]. Furthermore, uptaking more CoQ10 can lead to more vitamin E being available, which could have undesirable effects, particularly during the fish’s sensitive early stages. In this line, Hamre et al. [44] reported that high levels of vitamin E have been demonstrated to stimulate mortality and tissue lipid oxidation in marine fish larvae when there is inadequate vitamin C. Similarly, El Kertaoui et al. [45] and Hamre [46] reported that high amounts of vitamin E may serve as pro-oxidants.

The immune system and the antioxidant defense system of fish are inextricably intertwined [47]. The imbalance between the synthesis and disposal of reactive oxygen species triggers oxidative stress [48]. As key components of the antioxidant defense system, SOD, CAT, and GPx perform essential functions in eliminating excessive ROS [49,50]. The current data on antioxidant capacity in red tilapia reveal an improvement with CoQ10 treatments (C5 and C10), and this may be attributed to the intensity of CoQ10 in scavenging free radicals. Similar improvements in antioxidants have been previously reported with CoQ10 [24,25,37,38].

## 5. Conclusions

In conclusion, treating red tilapia fertile eggs with 5 mg/L CoQ10 improves the growth, intestinal structure, and antioxidant efficiency of the produced larvae. The current study’s findings support the use of CoQ10 in the hatching water in promoting the growth and well-being of the produced larvae, but they also raise the prospect of its inclusion in the diet of larvae throughout the weaning stage. Further studies on molecular reactions to environmental stimuli are crucial for the production of aquatic animals, particularly during the early phases.

## Figures and Tables

**Table 1:** Hatchability rate (%) and yolk-sac diameter (mm) (depth and width) of red tilapia larvae exposed to CoQ10 (0, 5, and 10 mg/L), during the first and second day post hatch (dph).

Treatments.	Hatchability %	Yolk-Sac Diameter (mm)			
		1 dph		2 dph	
		Depth (mm)	Width (mm)	Depth (mm)	Width (mm)
Control	38.00 ± 15.88	1.73 ± 0.15	1.94 ± 0.14 ^a^	1.86 ± 0.08	1.88 ± 0.23
C5	39.33 ± 6.11	1.72 ± 0.06	1.74 ± 0.04 ^b^	1.85 ± 0.13	1.79 ± 0.15
C10	54.67 ± 11.55	1.63 ± 0.07	1.77 ± 0.09 ^ab^	1.87 ± 0.25	1.63 ± 0.07

Means in the same column donated different superscripts are different (*p* < 0.05). Values are Means ± SD. dph, days post hatch.

**Table 2:** Standard length (SL) (mm), final total length (FTL) (mm), final weight (g), and survival rate (SR%) of red tilapia exposed to CoQ10 (0, 5, and 10 mg/L).

Treatments	SL (mm)				FTL (mm)	Final Weight 30 dph (g)	SR%	Deformed Fish%
	1 dph	2 dph	6 dph	30 dph				
Control	5.13 ± 0.15 ^a^	5.42 ± 0.56	7.22 ± 0.04	23.11 ± 1.84 ^ab^	30.00 ± 2.03 ^ab^	0.59 ± 0.06 ^a^	23.33 ± 7.02	-
C5	4.96 ± 0.19 ^ab^	5.59 ± 0.24	7.03 ± 0.33	25.33 ± 1.33 ^a^	32.67 ± 1.33 ^a^	0.53 ± 0.06 ^ab^	22.67 ± 11.02	-
C10	4.79 ± 0.02 ^b^	5.53 ± 0.09	7.39 ± 0.80	21.78 ± 1.35 ^b^	28.00 ± 0.88 ^b^	0.46 ± 0.02 ^b^	32.00 ± 2.00	2.08 ± 0.29

Means in the same column with different letters are different (*p* < 0.05). Values are Means ± SD. dph, days post hatch; Deformed fish%, the percentage of malformations was calculated from live or survived fish%.

## Data Availability

The datasets generated during and analyzed during the current study are available from the corresponding author on reasonable request.
